# Assessing the implementation of crustivoltaics in *trans* for the restoration of biological crust cover in remote sites

**DOI:** 10.1128/aem.00476-26

**Published:** 2026-06-17

**Authors:** Ana Mercedes Heredia-Velásquez, Ferran Garcia-Pichel

**Affiliations:** 1 Center for Fundamental and Applied Microbiomics, Biodesign Institute, Arizona State University 7864, Tempe, Arizona, USA; 2 School of Life Sciences, Arizona State University 7864, Tempe, Arizona, USA; Colorado School of Mines, Golden, Colorado, USA

**Keywords:** biocrusts, dryland restoration, remote site restoration, crustivoltaics, transplanted soils, trans-crustivoltaics

## Abstract

While biocrust restoration has gained relevance to combat increasing dryland soil degradation during the last decade, its scalability and ecological effectiveness remain limited because of the difficulty in producing large amounts of well-adapted, site-specific biocrust inoculum. Crustivoltaics, an approach where existing, local photovoltaic plants are used as large-scale microbial nurseries to produce biocrust inoculum, has been developed to overcome these barriers. Still, many degraded sites are in remote areas devoid of local solar farms. Here, we tested if crustivoltaics could be operated in a “*trans*” mode to generate compositionally appropriate inoculum for edaphically distinct or geographically distant sites. We assessed whether a facility in the Sonoran Desert built on sandy soils could be used to produce high-quality inoculum for a Chihuahuan Desert restoration target with gypsic soils, the sites having compositionally distinct native biocrusts. We transplanted target soils over native host soils, monitoring biocrust recovery for 16 months. Biocrusts developed on the transplanted soils grew at rates similar to those on native soil controls (0.70 ± 0.16 mg Chl *a* m^−2^ month^−1^), but only when left uninoculated. Their resulting microbial communities, however, did not faithfully resemble those native to the distant site. Instead, new biocrusts were compositionally intermediate between native and host sites, as a result of environmental filtering and community reshaping. Our study shows that while trans-crustivoltaics is possible in terms of the quantity of inoculum produced, it may be suboptimal in terms of quality if edaphic matching is not considered.

**IMPORTANCE:**

Increasing anthropogenic stress and climate change in dryland ecosystems have led to reduced soil fertility and increased dust emissions, which can affect human health. Because biological soil crusts help stabilize soils and maintain ecosystem function, they can be used to restore degraded dryland soils, where using solar power infrastructure as microbial nurseries to grow biocrust (crustivoltaics) is a promising strategy to scale up restoration. However, such infrastructure is not always available near degraded sites. In this study, we explore a trans-crustivoltaics approach, in which a solar facility is used to grow biocrust transplanted from a distant site to be restored. If successful, trans-crustivoltaics could expand the reach of biocrust restoration efforts.

## INTRODUCTION

During the last decade, the restoration of biological soil crust (biocrust) cover has emerged as one of the major thrusts in restoration ecology of arid lands, involving worldwide efforts to make this goal a reality. Biocrusts are cohesive, thin topsoil communities that rely on microbial or cryptogamic photosynthesis for primary production (1). They establish naturally and prominently on the topmost soils of drylands where vegetation is scarce, and are now recognized as being of great importance in dryland ecosystems, since they armor the soil against wind and water erosion (2–4), preventing the loss of organic carbon (5, 6). They also contribute to soil fertility through nitrogen and carbon fixation (7), where biocrusts fix about 3.9 Pg of carbon (8) and 9–14 Tg of nitrogen per year (9) globally. This is equivalent to 7% of the net primary production by terrestrial vegetation (8) and approximately 18% of global natural terrestrial biological nitrogen fixation (9).

Despite all the benefits that biocrusts bring to drylands, they are very fragile and highly vulnerable to physical disturbances such as livestock grazing, traffic, or human trampling (10). Currently, 12% of the continental surface is covered by biocrust, but due to increasing anthropogenic stress and climate change, its cover is expected to decrease by 25%–40% within 65 years (11). Because biocrusts grow slowly and only when soils are wet (12), their natural recovery can take decades to centuries (13–15), especially in hot and dry environments, though recent studies have shown faster recovery under certain conditions (16–18).

Biocrust restoration aims to develop strategies that promote the development of biocrust cover in crustless soils. Two main avenues are currently implemented, both involving the seeding of biocrust inoculants on degraded soils. In one, existing biocrusts salvaged from locations targeted for development are used as inoculants (19, 20). In the second, production of inoculum is attained by cultivation of appropriate biocrust organisms in laboratory-based or greenhouse nurseries (21–24). However, implementation has been constrained to rather small footprints, smaller than a few hundred square meters (25), due to either the lack of sufficient salvaged material or to excessive labor demands for inoculum cultivation.

Another major constraint, faced by nursery-based restoration in particular, is the need to tailor inoculum composition to resemble that of the local biocrust community. Since biocrust communities can differ widely from site to site. On the one hand, this increases the probability of successful establishment, as local community members will be genetically preadapted to local conditions (26). On the other hand, it serves to minimize the risk of introducing new species that could disrupt local ecosystem dynamics, notably pathogens (27). The optimization of inoculum composition can be complex, in that edaphic, as well as climatic factors, play a role in determining the composition of biocrust communities. Edaphic variables like soil texture, pH, mineral content, and parent material can be strong drivers of biocrust microbial community structure (28–30). Temperature, rainfall, aridity, and the levels of dust deposition also play a major role (31–34).

Recently, we developed crustivoltaics, a new approach for the generation of biocrust inoculum in which solar farms are used as *ad hoc* biocrust nurseries. It addressed the two major constraints above. Its high efficiency, low resource requirements, and little need for specialized management make it orders of magnitude more scalable than greenhouse or laboratory-based methods (25). Crustivoltaics has been proven to produce inoculum with microbial community composition that resembles that of natural biocrusts neighboring the solar facility. Thus, it is effective in promoting fast biocrust growth while keeping the desired community composition if implemented in locally existing photovoltaic facilities to restore neighboring soils (i.e., in “*cis*”). Despite the ever-increasing number and coverage of solar facilities in arid lands, an appropriately close facility may not be available close to restoration targets, or the targets, even if close to a facility, may have soil types different from those on which the solar farm is built, or both. The suitability of crustivoltaics to generate compositionally appropriate inoculum effectively in such cases (i.e., operating in “*trans*”) remains an untested possibility.

In this work, we evaluated trans-crustivoltaics for the worst-case scenario in which target sites differ from growth facilities in both climate and soil type. We chose a gypsum soil from the Chihuahuan Desert and attempted to grow similar biocrusts under a photovoltaic array on sandy soils of the Sonoran Desert. The choice was based on the fact that local and target biocrusts had clearly differentiated microbial composition. We then monitored the fate of transplanted plots and appropriate controls, both in terms of biomass development and community composition, for a period of two years.

## RESULTS

### Biocrust development

**Fig 1:**
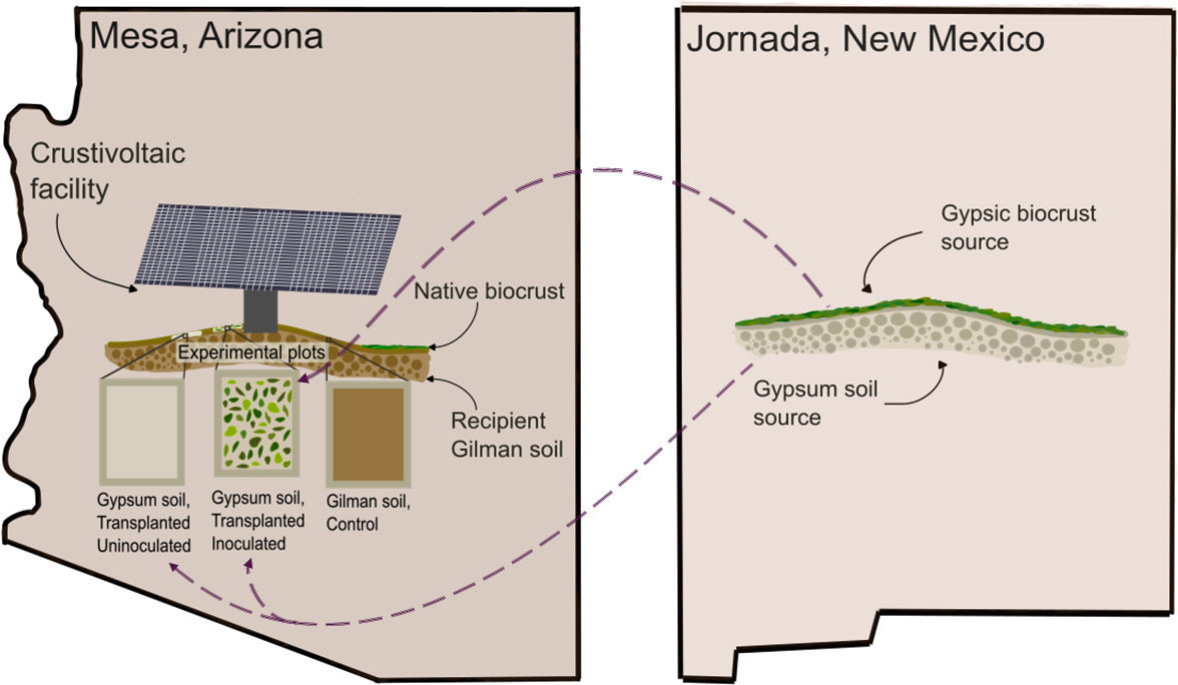
Experimental design showing the relocation of soil from New Mexico’s Chihuahuan Desert to Arizona’s Sonoran Desert. Dashed arrows indicate the transplant of soil and inoculum. Three types of experimental plots were set up, two with transplanted soil (either inoculated with Chihuahuan gypsum soil biocrusts or left uninoculated) and one with local native soil as control, left uninoculated.

**Fig 2:**
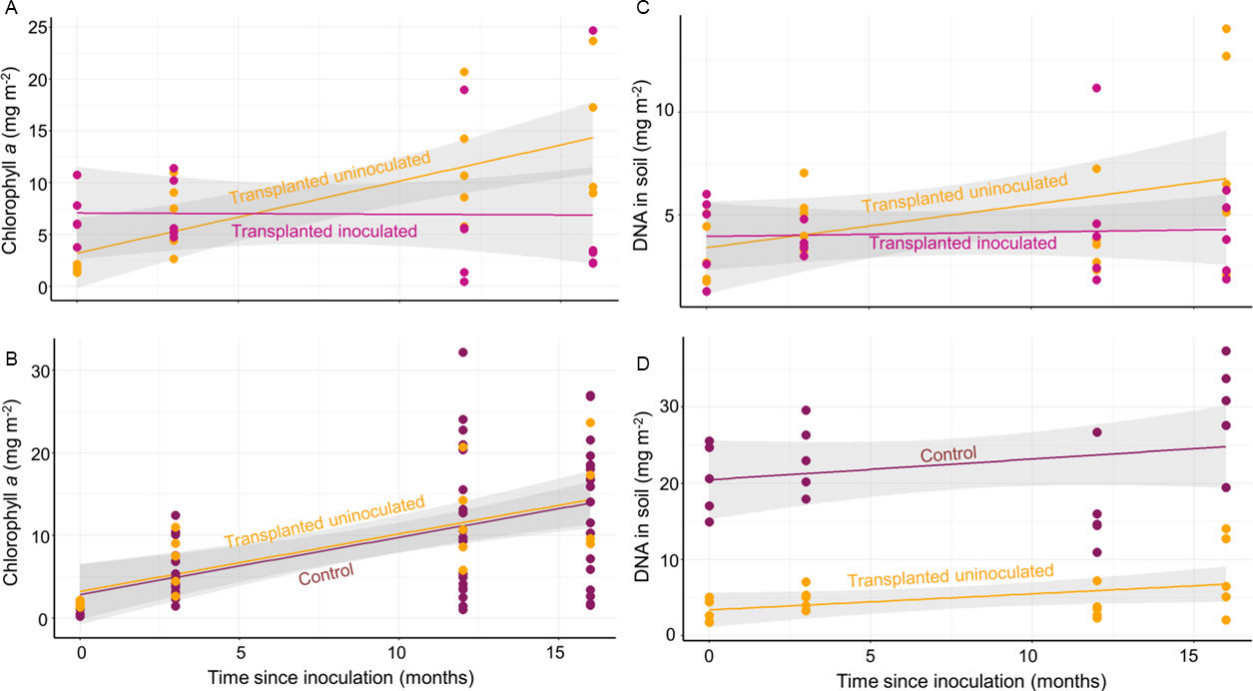
Cyanobacterial and microbial community growth in experimental plots. Left plots (**A and B**) are for Chl *a*, and right plots (**C and D**) for community DNA. All panels share the same x-axis; x-axis labels are only displayed on lower panels to avoid redundancy. Treatments are color coded. Values for all replicate plots (*n* = 5 per time point and treatment, unless otherwise stated) are included, with respective linear regression fits against time as color-matched lines. Gray shaded areas depict the 95% CI for the regression slopes. Statistics for Chl *a*: transplanted inoculated (slope= −0.01 mg [Chl *a*] m^−2^ month^−1^; *R*^2^ = 2 × 10^−4^), transplanted uninoculated (slope = 0.70 [Chl a] m^−2^ month^−1^; *R*^2^ = 0.52), control (slope = 0.69 [Chl *a*] m^−2^ month^−1^; R^2^ = 0.25), slope of transplanted uninoculated was greater than that of transplanted inoculated (*P* = 0.01), and no different from control (*P* = 0.99). Statistics for DNA: transplanted inoculated (slope = 0.02 mg [DNA] m^−2^ month^−1^; *R*^2^ = 4 × 10^−3^), transplanted uninoculated (slope = 0.21 mg [DNA] m^−2^ month^−1^; *R*^2^ = 0.18), control (slope = 0.27 mg [DNA] m^−2^ month^−1^; *R*^2^ = 0.06), slope of transplanted uninoculated was only marginally greater than that of transplanted inoculated (*P* = 0.16), and no different from control (*P* = 0.82). Slopes were compared using analysis of covariance (ANCOVA). In control plots, 1–4 samples per plot were analyzed per time point.

We set two types of experimental 1 m^2^ , initially crustless, plots under the shade of solar panels. An approximately 1 cm thick layer of soil from the Chihuahuan site was laid over the existing soil. In one type, plots were inoculated with crumbled, native biocrust from the target site (“transplanted, inoculated”), and in the other, the soil plot was left uninoculated (“transplanted, uninoculated”). As “controls,” crustless plots were also set up with local soil only and left uninoculated (Fig. 1). To assess biocrust development, we tracked biomass using two different parameters: chlorophyll-*a* (a proxy for cyanobacterial biomass) and total community DNA (a proxy for overall microbial biomass) within the top 1 cm of soil. Chlorophyll *a* (Fig. 2A) increased consistently in the transplanted uninoculated plots, but surprisingly, it remained statistically unchanged in transplanted inoculated plots (only one of five plots showed a significant increase, while the other four decreased; Fig. S1). The average Chl *a* content in the transplanted uninoculated plots increased by fivefold during the study period, with a rate of 0.70 ± 0.16 mg Chl *a* m^−2^ month^−1^, but the average for transplanted inoculated plots had virtually no change (0.01 ± 0.21 mg Chl *a* m^−2^; not significantly different from zero). The two rates were significantly different (*P* = 0.01) when analyzed as treatment averages; however, when growth rates were estimated for each plot independently, the difference was only marginally significant (*P* = 0.07; Fig. S1). This implies that cyanobacteria grew on the uninoculated foreign substrate but not if inoculated. These results were unanticipated and surprising, though their statistical support varies depending on the approach of analysis. Both cyanobacterial growth rates and final yield in the transplanted uninoculated plots were statistically indistinguishable (*P* = 0.99) from those obtained in the control plots on native soil, either when growth rates were averaged or treated independently (Fig. 2B and Fig. S1). This indicates that biocrusts developing on transplanted soil could recover as fast as those under typical “*cis*” operation. Acknowledging that the results of the transplanted inoculated plots remain a concern, the results on the transplanted uninoculated speak well for the potential of trans-crustivoltaics.

The dynamics of community DNA (Fig. 2C and D) followed similar trends to those obtained with Chl *a*, though the differences, both with time and between treatments, were more moderate. This is perhaps not surprising as overall community biomass typically trails that of its primary producers (35, 36). Figure 2C shows that the average DNA content increased in transplanted uninoculated plots by twofold, but not at all in transplanted inoculated plots. Statistically, the slope for a linear trend was marginally significant with a *P* = 0.06 for the uninoculated treatment. However, the growth rates measured by community DNA were only poorly significant by treatment (*P* = 0.158). Again, here, the dynamics in DNA concentration of the transplanted uninoculated were indistinguishable from those in the control plots (Fig. 2D; *P* = 0.82), with control plots showing an average yield of 1.5-fold.

### Dust deposition role in biocrust development

**Fig 3:**
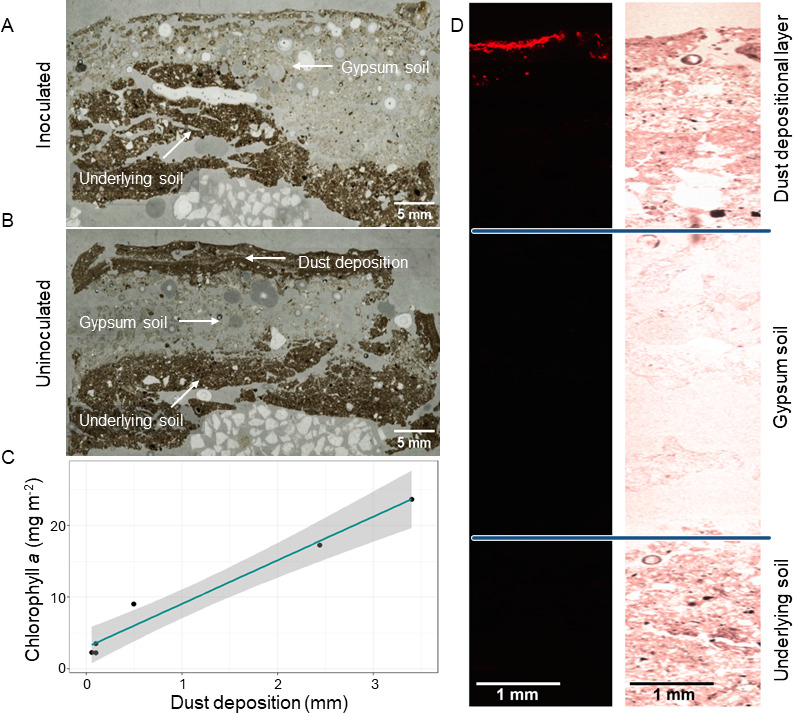
Relationship between dust deposition and cyanobacterial growth. (**A and B**) Polarized light photomicrograph of a soil thin section from a transplanted inoculated and transplanted inoculated plots, as labeled. (**C**) Correlation between dust deposition and Chlorophyll *a* yield among plots. Gray shaded area delimits the 95% CI for the slope, and the solid lines represent the best-fit linear model. Statistics for Chl a per dust deposition: (slope = 6.08 [Chl a] m^−2^ dust deposition mm^−1^; R^2^ = 0.97; *P* = 4 × 10^−4^). (**D**) Fluorescence (left) and brightfield (right) photomicrographs of a vertical thin section of a transplanted uninoculated plot. Red autofluorescence in the left indicates cyanobacterial biomass. Albedo in the brightfield images denotes soil composition (gypsum has high albedo, and newly deposited or underlying soil has low albedo).

During our sampling, we observed an apparent but unpredicted phenomenon that may have influenced the growth patterns described in the previous paragraph: transplanted uninoculated plots appeared to have decreased in albedo, turning more tan (not greener) while transplanted inoculated plots did not, as if they had received more dust deposition. To determine if this was the case, we prepared petrographic thin sections of resin-embedded topsoil samples for detailed examination. These thin sections (Fig. 3A and B) indeed confirmed marked differences in dust deposition. Newly deposited layers of dust, 0.5–3.4 mm thick, were plainly visible in the transplanted uninoculated plots but much thinner or non-existent in the transplanted inoculated plots. This difference was patent even in plots that were adjacent (Fig. S2), so that a differential effect driven by relative plot location can be discounted. Incidentally, the single inoculated plot that grew was surrounded by transplanted uninoculated and control plots that exhibited lower chlorophyll-*a* yields, so its unorthodox behavior within the treatment was likely not caused by position either. We investigated the potential functional consequences of the dust deposition by assessing two different factors: (i) the relationship between total dust deposition and chlorophyll-*a*, and (ii) the location of the cyanobacterial growth within the soil at the microscale. We found a strong correlation between chlorophyll-*a* yields at the end of the experiment and the intensity of dust deposition (Fig. 3C), gauged by the thickness of the top depositional layers with an *R*^2^ of 0.97 and a *P* < 0.0004. Furthermore, observations on the thin sections under fluorescence microscopy revealed that the red autofluorescence typical of cyanobacterial pigments collocated largely to the newly deposited layers themselves, tightening the link between deposition and growth (Fig. 3D). These results could indicate that dust deposition promoted biocrust growth by providing a better habitat or that biocrust development was conducive to trapping and binding of fugitive dust particles. Evidence in support of either of the two alternatives exists in the literature (32, 37–39).

### Microbial community composition

**Fig 4:**
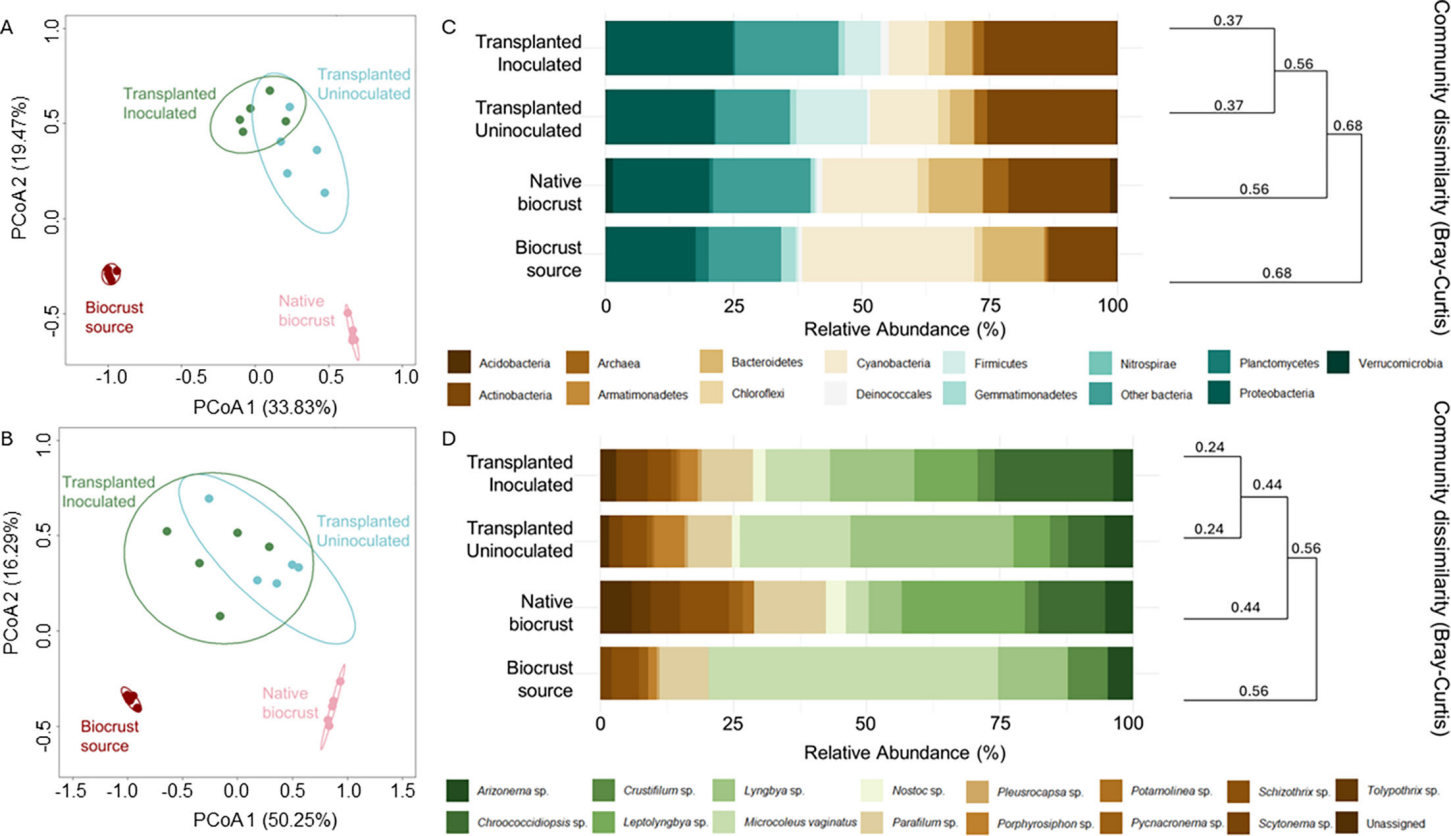
16S rRNA gene-based microbial community composition of source biocrusts and experimental plots after 16 months of development. (**A and B**) Principal coordinate analyses based on Bray-Curtis dissimilarity (*n* = 5), for bacterial amplicon sequence variant (ASV) (**A**) or cyanobacterial ASVs (**B**). Ellipses indicate 95% confidence areas by treatment. (**C and D**) Taxon relative abundance (average of *n* = 5 independent determinations) for bacteria resolved at the phylum level (**C**) and for cyanobacteria resolved at the genus level (**D**). Overall, Bray-Curtis dissimilarity clustering is shown to the left.

We also followed the fate of the microbial communities as they developed, with particular attention to those in the new settings of a foreign and distinct soil substrate. The two potential source communities were those of biocrusts native to the solar farm site and those native to the target site of origin. Bacteria from the target site may have been carried over as propagules within the transplanted bulk soil (i.e., as cyanobacterial seed banks [16]) or introduced with active inoculation. Principal coordinate ordination of their 16S rRNA-based community composition revealed that these two end-member communities were quite different and easily recognizable, either by considering all bacteria or just cyanobacteria (Fig. 4A and B, respectively). While the communities developing on transplanted plots were self-similar among replicates, they underwent clear shifts from either one of the end-members. These ordinations were robust and explained 53% of the variation for bacteria and 67% of the variation for cyanobacteria. Interestingly, even in the inoculated plots that had shown no net growth, the communities were not in stasis, as shifts in community composition from that of the inoculum had taken place.

Relative abundance plots can be used to gain an idea of the specific changes that these community shifts entailed (Fig. 4C and D). These plots include hierarchical clustering to aid in assessing community similarity. Considering all bacteria, transplanted plots on average had self-similar communities (37% dissimilarity) but a 56% dissimilarity with the native biocrust of the crustivoltaics site and were 68% dissimilar to the source gypsum biocrusts. Specifically, the transplanted plots had a lower relative abundance of cyanobacteria compared to the other two soils, and a higher relative abundance of Firmicutes, probably as a result of being less mature in the ecological succession, a stage in which cyanobacteria tend to dominate clearly. When considering only cyanobacteria, the communities of the two types of transplanted plots were again most self-similar (24% dissimilarity). For cyanobacteria, the transplants resembled the biocrust source more (44% dissimilarity) and the native biocrust least (56% dissimilar; Fig. 4D), though these differences may not be significant.

When relative abundance was analyzed by plot (Fig. S3), inoculated plots exhibited heterogeneous community composition among replicates and did not cluster. In fact, some inoculated replicates showed greater similarity in composition to uninoculated communities, while others were more similar to the biocrust source. The inoculated plot that clustered most closely with the uninoculated plots was the single one that grew. It was characterized by a higher relative abundance of *Lyngbya* sp., whereas this taxon was less abundant in other inoculated replicates.

**Fig 5:**
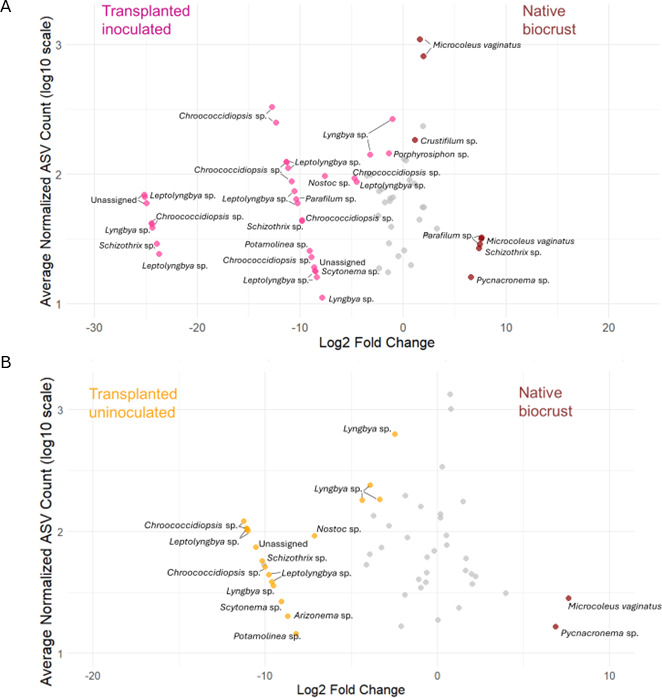
Differential abundance of cyanobacterial ASVs between native, local biocrust, and transplanted plots. (**A**) Comparison with transplanted, inoculated plots. (**B**) Comparison with transplanted, uninoculated soils. ASVs showing significant differences are colored, nonsignificant in gray. Low-abundance ASVs (<5 counts in at least two samples) were excluded.

To determine which specific cyanobacterial taxa drove the differences between the transplanted plots and the native biocrust, we performed a differential abundance analysis based on cyanobacterial amplicon sequence variants (ASVs; Fig. 5A). ASVs corresponding to *Chroococcidiopsis* sp. were significantly enriched in the transplanted inoculated plots. In contrast, ASVs corresponding to *Microcoleus vaginatus* were significantly enriched in the control biocrusts. These compositional differences are consistent with the relative abundance observed in Fig. 4D. When comparing the transplanted uninoculated plots and the native biocrust, a broadly similar pattern was revealed (Fig. 5B). The ASVs that were more predominant in the transplanted uninoculated plots corresponded to *Lyngbya* sp. and *Chroococcidiopsis* sp. In contrast, in the native biocrust, the most predominant ASVs were those that corresponded to *Microcoleus vaginatus* and *Pycnacronema* sp. Although both transplanted plots were enriched with similar cyanobacterial taxa, the relative abundance of these assemblages differed between the uninoculated plots and the inoculated plots. *Chroococcidiopsis* sp. was more dominant in the inoculated plots, and *Porphyrosiphon* sp. (Fig. S4) was more dominant in the uninoculated plots.

## DISCUSSION

### Biocrust growth dynamics in trans-crustivoltaics

Our results demonstrate that biocrusts can develop in a trans-crustivoltaics setting with efficiency indistinguishable from those carried out concurrently in *cis*. However, two of our findings, which remain somewhat puzzling, add cautionary qualifications to that statement.

On the one hand, and contrary to what we have seen previously when biocrusts were grown in cis-crustivoltaics (25), soil inoculation did not result in increases in rate or yield. It is not rare in biocrust restoration that inoculation fails to provide improvements over non-inoculated soils (24, 40–42). Factors like active soil erosion, resource limitations, and physiological stress (13, 17) have been advanced as explanations. However, we could not find another case in the literature where inoculation made outcomes worse, in that inoculation seemed to have prevented growth completely in treatment averages, although there was growth in one plot, and four showed net declines. This remains mysterious to us. Since inoculation involved spreading inoculum over the very soil surface, this inoculum may have been subject to harsh environmental conditions of solar insolation or surface abrasion. Notably, growth did not proceed, either based on the inoculum itself or from naturally existing microbial seed banks that support biocrust recovery without interventional inoculation (16). On the other hand, the close, if unforeseen, link between growth and dust accretion adds another layer of uncertainty. Potentially, inoculation might have resulted in physical changes in the soil surface that prevented dust trapping and, indirectly, biocrust growth. It is, however, hard to explain that adding biocrust particles that impacted only 25% of the surface could have had such a clear negative and widespread effect on dust trapping. We recognize that while there is a potential for trans-crustivoltaics operations in terms of biocrust growth, some of the new questions that our study opened should be further clarified before implementation.

The fact that the growth of the microbial communities, as determined by whole community DNA, seemed to have trailed that of cyanobacteria was not so surprising, as the whole community is dependent on cyanobacterial primary production, and the relatively short experimental period may not have allowed for this production to trickle down to other trophic layers in the community, which delayed colonization by heterotrophic bacteria.

### Microbial community composition in transplanted soils

All transplanted plots, regardless of treatment, harbored a similar community composition, which was intermediate between those typical of the native biocrusts growing on the host and origin sites. This indicates that while community members typical of the gypsic soils of origin could grow in the host site, they had to compete with microbes that are typical of the host site, likely a consequence of the interaction of climate (Sonoran) with edaphic character (gypsic). This is consistent with studies showing the local influence on community composition of biocrusts (28, 29) and that soil substrate exerts selective pressure on microbial community assembly (43–46). Specifically, gypsum soils are reported to harbor higher abundances of Actinobacteria and Proteobacteria with comparatively fewer Cyanobacteria (47) than other soils. It is also consistent with known cyanobacterial taxa-specific preferences: the observed increase in relative abundances of *M. vaginatus*, a species commonly dominant in sandy and silt soils and absent or very minor in gypsic soils (43).

We note that community shifts with such intermediate outcomes took place regardless of net growth, since they also occurred in the transplanted inoculated plots. In other words, communities that failed to grow were still actively undergoing community shifts and were thus not static. It is a possibility that in the absence of dust accretion, these communities suffered from nutrient limitation and would have been much less productive than those where dust inputs were more marked.

In practical terms, it is then possible that the inoculum developed in *trans* would be appropriate for re-inoculation at the target site, as it contains the right community members, but only sub-optimally so, in that it also contains microbes typical of foreign sites. These results strongly suggest that an eventually optimized trans-crustivoltaics inoculum production should consider edaphic matching in the experimental design. By doing so, one would maximize the compatibility and establishment of the inoculated biocrust material (48).

### Dust role in biocrust growth

The clear correlation between biocrust growth and dust deposition, along with the high presence of cyanobacteria within the depositional layer (Fig. 3), points to the importance of aeolian dust for biocrust development. In arid lands, where soils are often nutrient-limited, dust can be a good source of nutrients such as phosphorus, potassium, magnesium, calcium, and iron (49, 50). Dust deposition can also alter the texture of the soil surface, influencing water infiltration and retention patterns (37), and it can also provide a matrix that acts as a refugium for the establishment of cyanobacteria migrations *within* the soil (as opposed to *on top of* it, as is the case with inoculants), leading to faster growth (32). In addition to these physical and chemical effects, microbial deposition through dust may also be contributing to microbial colonization. It has been shown that dust particles carry viable microorganisms across arid landscapes, and that they are particularly enriched in Proteobacteria and Actinobacteria, although biocrust organisms seem rare (51). During high-deposition periods, airborne communities may contribute up to 10^8^ bacterial 16S rRNA gene copies per cm^2^ month^−1^, which is comparable to the microbial biomass present in biocrusts (1). Any or all of these factors may be behind a purported beneficial effect of dust on biocrust growth. The alternative driver of this correlation is that the growth of cyanobacteria promotes the trapping of deposited dust particles through their investment in exopolysaccharides, which has been experimentally demonstrated as well (52). We cannot fully disentangle these two explanations from our data. But given that the transplanted inoculated plots also contained cyanobacterial populations, and yet did not retain much dust, the former seems more likely. The effects described here, however, clearly add to the mounting evidence that dust deposition plays a central role in the ecology of biocrusts, one that has likely not received sufficient attention in restoration attempts. Because dust inputs will tend to be sourced in local soils, the effects of dust clearly represent a complication likely to be important in trans-crustivoltaics.

### Trans-crustivoltaics as a restoration strategy

Our findings raise important considerations for operating crustivoltaics in *trans*. Pending further clarification, the current conclusion is that surface inoculation may prevent, and certainly does not facilitate, the establishment of biocrust microbial communities in this mode, unlike the advantages seen in the *cis* mode. Even if growth is achieved, the communities that developed diverged from those in the intended target site, even when retaining significant proportions of the microbes that were dominant there. For that reason, the inoculum obtained from trans-crustivoltaics may be at best sub-optimally adapted to the specific conditions of the restoration site, potentially limiting its establishment (53). When interpreting these results in a wider context, we should also consider the choice of soils for transplant. In our case, this was partly driven by the goal of having communities and edaphic properties as different as possible. In many applications, such differences may be less marked, and thus, microbial community shifts more subdued. In any event, the experiment certainly highlights limitations of the trans-approach. One could potentially ameliorate these community effects by better matching the edaphic and climatic conditions between the facility and the restoration target site. The conditions might not need to be identical or geographically close, but they should be more similar than in our initial study.

## MATERIALS AND METHODS

### Experimental design

To evaluate the potential of trans-crustivoltaics, we set up experimental plots under photovoltaic panels in a facility in Mesa (AZ) in the Sonoran Desert (Fig. 1). This involved scalping off all existing biocrusts in all plots. In some plots, a layer of gypsum soil from the Chihuahuan Desert (Jornada Basin, NM) was laid down. Half of the plots received an inoculum of crumbled biocrust collected in the gypsum soil (“transplanted inoculated”), and half of these plots remained uninoculated (“transplanted uninoculated”). As controls, some plots received no foreign soil nor inoculum. Their fate was monitored for 17 months. Plots were placed randomly (see Fig. S2).

### Experimental plots

Biocrust from four different locations representing two soil types was collected using Petri plates and comprising the top 1 cm of soil. Three of these soil samples were obtained from gypsum-rich soils and one from Gilman sandy soil. The first gypsic soil was collected in the Jornada Experimental Range (32°41'36.9"N, 106°46'41.9"W) near Las Cruces, New Mexico, and two were collected from (32°44'55.1"N, 106°11'41.8"W) near Alamogordo, New Mexico. Although the last two samples were taken from the same general area, they differed in physical structure. One consisted of loose sand, while the other was a consolidated, rock-like material. Gilman soil samples were collected from the Poly Ground Mount 2 solar facility in Mesa, Arizona (25). Microbial community composition was analyzed using principal coordinates analysis (PCoA) based on Bray-Curtis dissimilarity (Fig. S5). Among the three gypsum-rich sites previously sampled, Jornada soil was selected for experimental use due to its logistical convenience for collection and transport.

Gypsum soils from Jornada, New Mexico, were collected after the removal of biocrusts. The upper 3–5 cm of surface soil containing the biocrusts was removed using a shovel, and bulk soil was then collected and transported to the solar facility in Arizona. Soil transplantation was performed to assess the potential of tailoring soil type-specific biocrust communities under the influence of photovoltaic panels. One square meter plots in the solar farm in Arizona were cleared of existing biocrust, and corners were staked using rebar for ease of recognition. Plots were either left to recover naturally (control, *n* = 5) or a gypsum soil layer was added (*n* = 10). To create the gypsum soil plots, a 1 cm thick layer of gypsum bulk soil was laid down, representing the typical depth of the cyanobacterial biocrust zone of influence. Half of the gypsum plots were left uninoculated, and the other half were inoculated with biocrust collected from the site of origin. All experimental plots were wetted immediately after the soil was added, and immediately after inoculation was carried out. Biocrust used for inoculation had been previously crumbled into mm-large pieces and then manually sown in the plots to reach an initial average chlorophyll-*a* concentration of 7 mg m^−2^, which corresponded to 25% of the areal concentrations normally found in the biocrusts collected. This process took place in November 2023 to take advantage of the winter rain season. Plots were monitored for 16 months to encompass two winter growth seasons.

### Assessment of biocrust development

Biocrust development was monitored at four key points: post-inoculation (November 2023), first post-winter rains (February 2024), post-monsoon season (November 2024), and second post-winter rains (March 2025). At each sampling time, five 6.8 cm^2^ samples of the top cm of the soil were taken randomly for each experimental plot. The five samples were then homogenized and used for chlorophyll-*a* (chl-*a*) determinations and DNA quantification, both proxies for biomass of cyanobacteria and all microbes, respectively.

A subset of ~1 g of soil was used for the pigment extraction. Soil sample was ground with a mortar and pestle for 3 min in 4 mL of 90% aqueous acetone (26). Subsequently, slurries were transferred to 15 mL Falcon tubes, vortexed for 30 s, and incubated in the dark for 24h at 4°C to allow extraction. After incubation, slurries were vortexed for 30 s and centrifuged for 7 min. Chl-*a* content in the supernatant was determined spectrophotometrically using a Shimadzu UV-2600i spectrophotometer. Interference from scytonemin and carotenoids was discounted using trichromatic equations (54). Chl-*a* values were converted to the full core and expressed as mg m^−2^ based on previously determined core mass and surface area.

Prior to DNA extraction, 0.25 g of soil was treated with a 100 mM EDTA solution to dissolve gypsum and release microbial cells (55). The resulting suspension was vacuum filtered through a glass fiber filter to collect the microbial biomass along with the residual non-gypsum particles. To remove any remaining EDTA, biomass and residual particles were washed three times by resuspending the pellet in sterilized 18 μΩ Milli-Q water, followed by filtration. Genomic DNA was extracted directly from these filters by inserting them into the bead-beating tubes of the FastDNA Spin kit for soil (MP Biomedicals). Extractions were then performed following the manufacturer’s protocol. Triplicates of each sample were mixed and then quantified. The total DNA was fluorometrically quantified using a Qubit dsDNA HS (High Sensitivity) assay kit (Invitrogen) following the manufacturer’s protocol.

To quantify biocrust development over time, linear regressions were performed for chl-*a* and total DNA. The slopes of these regression lines represented the growth rates of biocrust at the different experimental plots. Differences in the growth rate among treatments were evaluated using an analysis of covariance using R (version 4.5.0) (56).

### Impact of dust deposition and biocrust development

Three-centimeter-deep soil samples were carefully cored from the experimental plots, using plastic cylinders and a spatula. Soil cores were stabilized by embedding them in a water-based resin (EpoFix, Struers APS, Ballerup, Denmark). Subsequently, the soils were sent to Spectrum Petrographics (Vancouver, Washington, USA) for commercial thin sectioning (32). Polarized light micrographs were taken from the thin sections and were further analyzed using ImageJ (57) to measure the soil depositional layer. Thin sections were then examined under a fluorescence microscope to identify cyanobacteria through their photopigment autofluorescence, using a yellow excitation filter and a red bandpass emission filter.

### Community composition

Microbial community composition was characterized through next-generation sequencing of 16S rRNA genes. Universal bacterial primers 515F and 806R were used to amplify the V4 region of 16S rRNA genes according to the Earth Microbiome Project (58). Sequencing yielded raw FASTQ files that were later processed using Qiime2 v2024.10 with the Qiime2-amplicon environment (59). ASVs were obtained using the DADA2 plugin (60), and preliminary taxonomic assignments were made using Greengenes 13_8 database (61). Cyanobacterial and non-cyanobacterial sequences were separated into two feature tables, and the cyanobacterial features were further curated via Cydrasil3 (62).

Subsequent analyses were conducted in R (version 4.5.0) (56). Bray-Curtis dissimilarity matrix was performed using the vegan package (63), which was later used to prepare a PCoA and a hierarchical clustering. PCoA was generated for both the whole microbial community and cyanobacteria subset to visualize the overall difference in community composition. Hierarchical clustering was represented as a dendrogram using the ggdendro package (64). The dendrogram was used to order samples in the relative abundance barplot, supporting the interpretation of patterns in taxonomic composition across the experimental plots. Relative abundance barplots and MA plots were prepared using ggplot2 (65). To identify taxa driving the community differences among the experimental plots, a differential abundance analysis was carried out using DESeq2 (66).

## Data Availability

Molecular data for microbial analyses are freely available through NCBI under BioProject number PRJNA1395609.

## SUPPLEMENTAL MATERIAL

Supplemental material aem.00476-26-s0001.docx Fig. S1 to S5.

ASM does not own the copyrights to Supplemental Material that may be linked to, or accessed through, an article. The authors have granted ASM a non-exclusive, world-wide license to publish the Supplemental Material files. Please contact the corresponding author directly for reuse.
