# Supplementary material for: Assessing the implementation of crustivoltaics in trans for the restoration of biological crust cover in remote sites
Source: Appl Environ Microbiol. 2026 Jun 17;92(7):e00476-26. doi: 10.1128/aem.00476-26 (PMC13390386; doi:10.1128/aem.00476-26)
Supplement: Supplemental material — Fig. S1 to S5. [file aem.00476-26-s0001.docx]

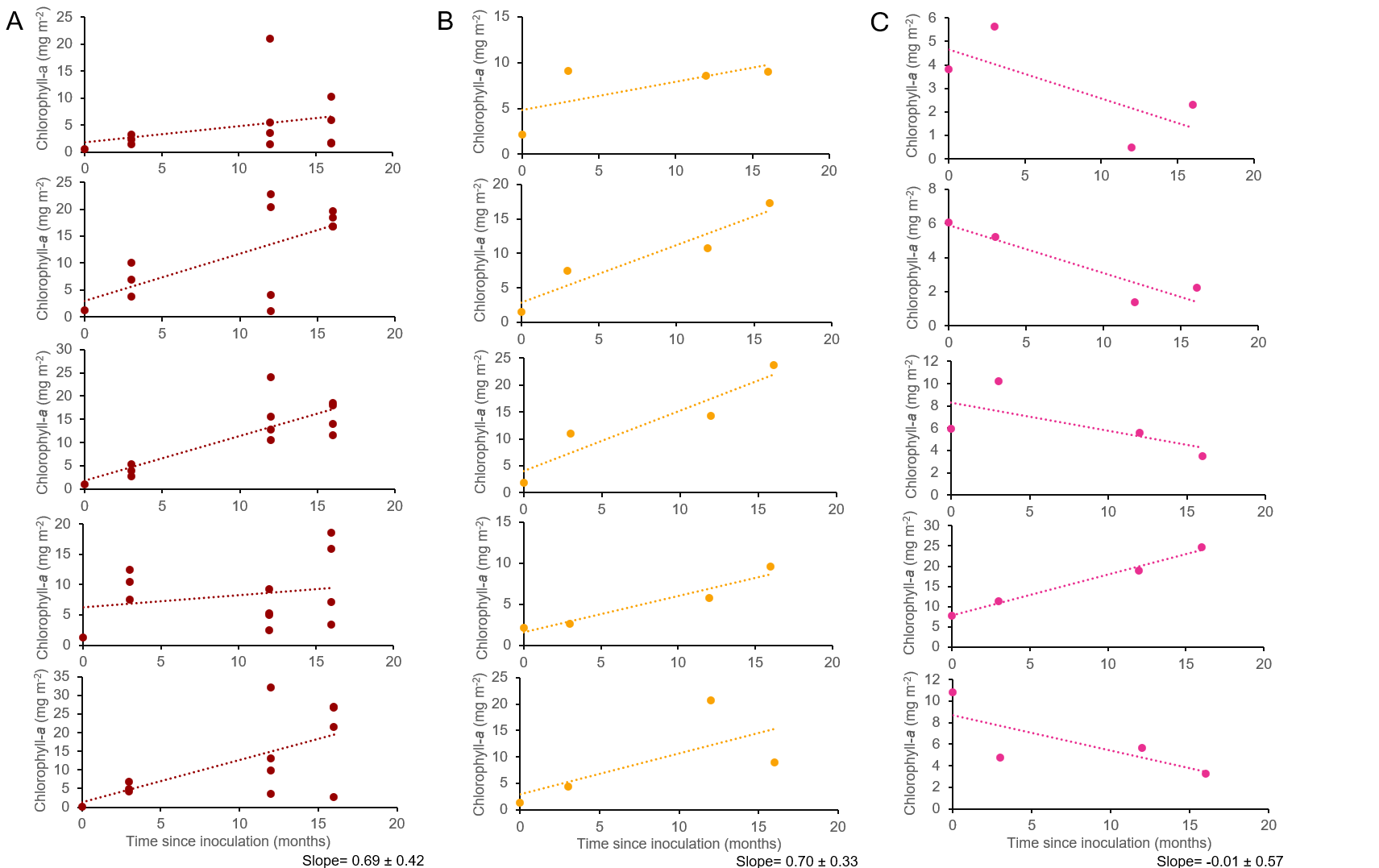


Figure S1: Cyanobacterial growth in experimental plots. All panels share the same x-axis; x-axis labels are only displayed on the bottom graphs to avoid redundancy. Panels are arranged in columns by treatment: (A) control plots (maroon), (B)transplanted uninoculated (yellow), and (C) transplanted inoculated (pink). All obtained values are shown (n = 1 per time point and treatment, unless otherwise stated), along with linear regression fits. Slopes of each replicate per treatment were compared through ANOVA, which indicated significant differences among treatments (P= 0.04). A Tukey post hoc test showed that transplanted uninoculated plots were not different from the control plots (p=0.99), and transplanted inoculated plots were only marginally significantly different from both the control and transplanted uninoculated plots (P= 0.07).


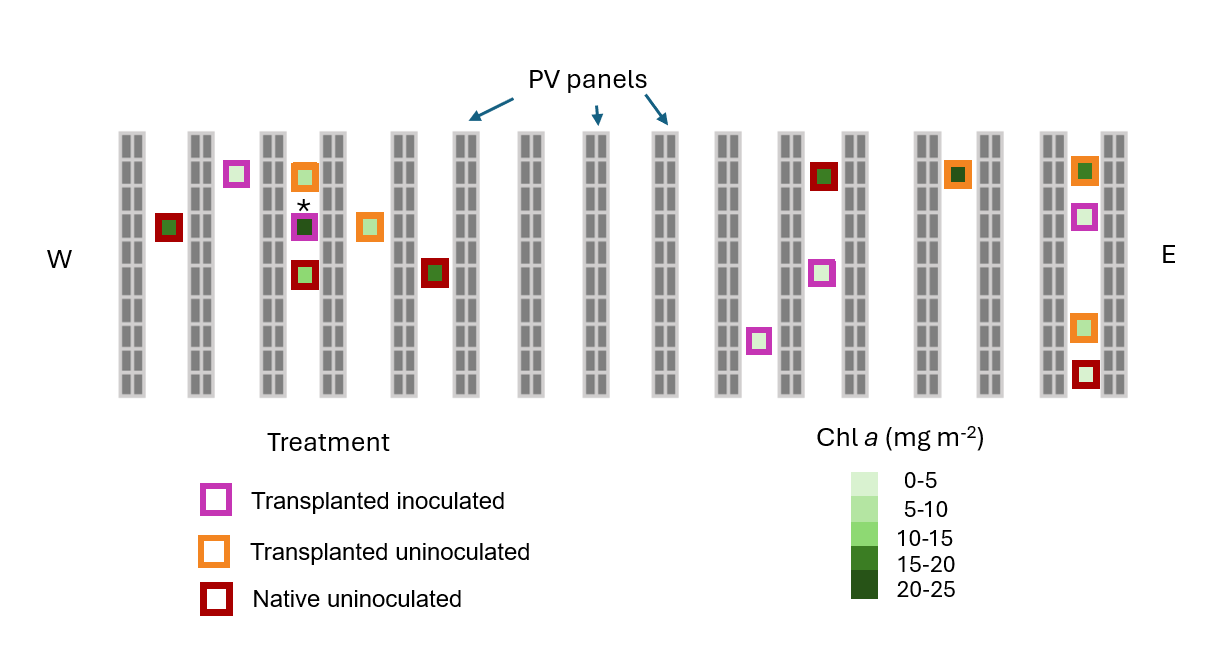


Figure S2: Placement of experimental plots at the Poly Mount 2 solar farm. Squares mark the location of the experimental plots in relation to the layout of PV rows. Square outline color represents the treatment of each experimental plot, while fill color represent biomass levels obtained. The darker the fill color, the higher biomass levels. Asterisk on top of the square marks the only transplanted inoculated plot that grew.


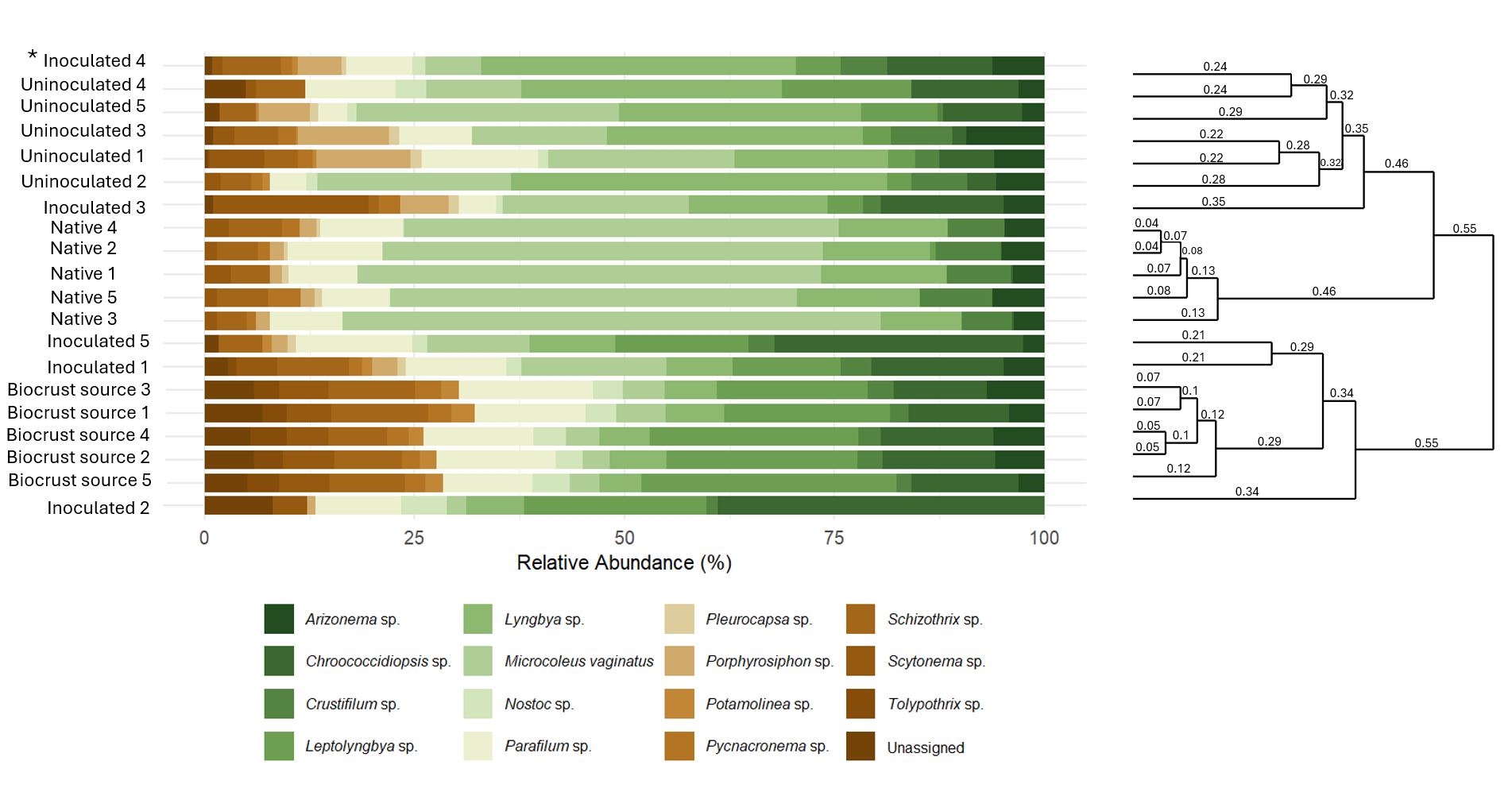


Figure S3: 16 S rRNA gene-based microbial community composition for each experimental plot. Cyanobacterial relative abundance, resolved at the genus level, is shown. Hierarchical clustering based on Bray-Curtis dissimilarity clustering is shown on the left. A single transplanted inoculated plot that exhibited growth is marked with an asterisk.


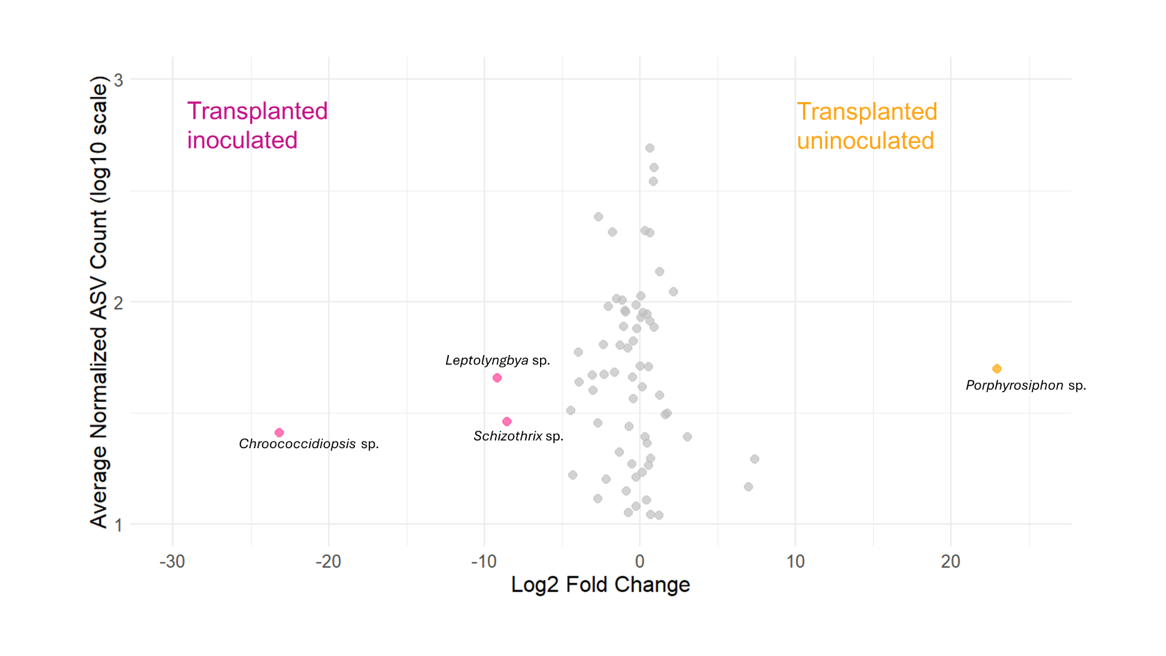


Figure S4: Differential abundance of Cyanobacterial ASVs between transplanted uninoculated plots and transplanted inoculated plots. Statistically significant ASVs are colored and nonsignificant ASVs are represented in grey. Low-abundance ASVs were excluded from analysis by retaining only those present with counts greater than 5 in at least 2 samples.


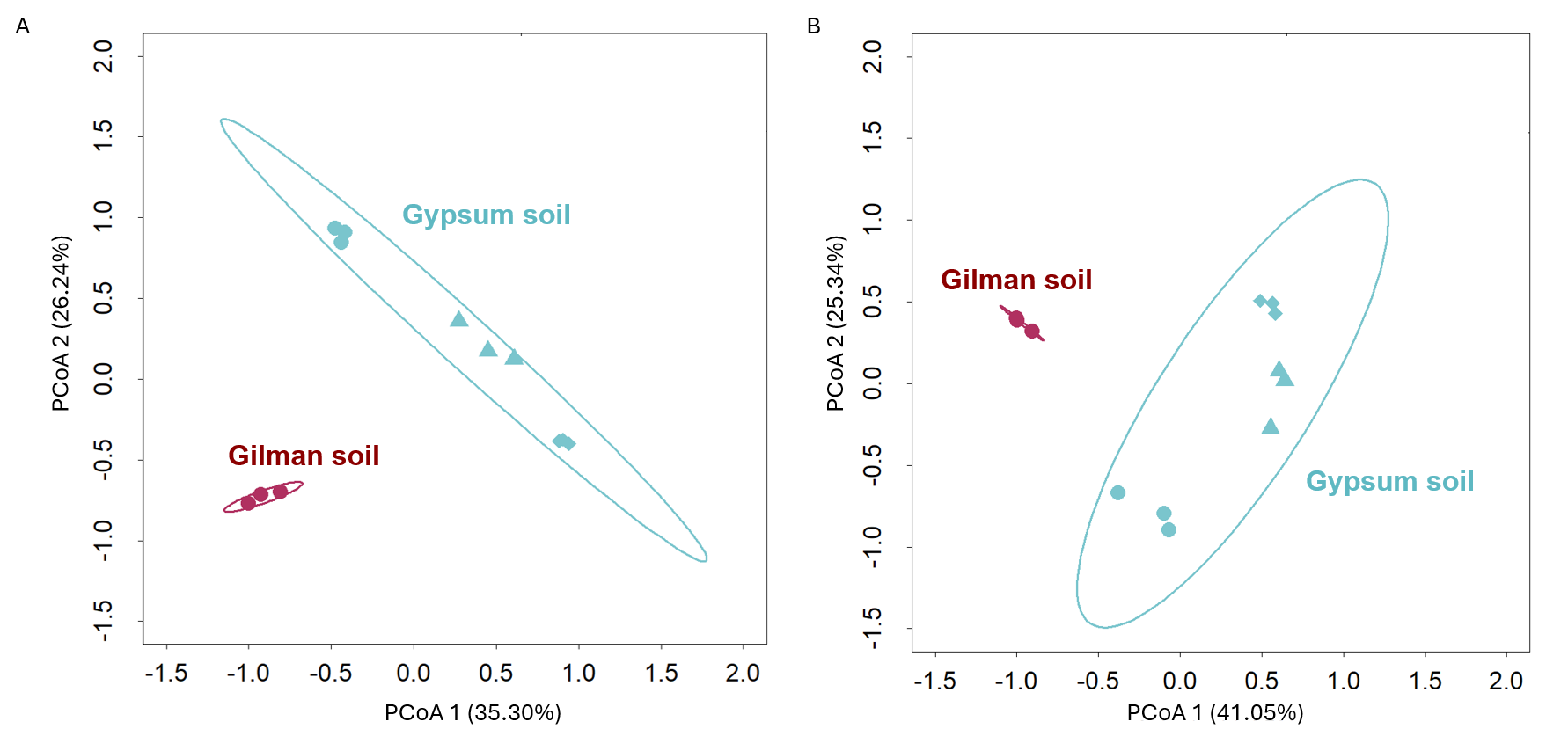


Figure S5: Principal Coordinate Analysis based on Bray-Curtiss dissimilarity of microbial communities found in native soils in the crustivoltaics facility (Gilman Soils) and gypsic soils, as preliminary analyses before choosing a source soil for experimentation (A) Analysis based on all bacterial ASVs (B) analysis based only on ASVS corresponding to Cyanobacteria. Circles represent soils taken from Alamogordo that had a sandy texture, triangles represent soil samples taken from Alamogordo that had a rocky structure, and squares represent samples taken from Jornada Basin. The dots represent the community composition in each soil sample (n=3). Ellipses indicate 95% confidence area for the respective soil community. All sources differed sufficiently from Gilman Soils, and Jornada samples were chosen for convenience.
